# Integrating Health Policy Education Into an Undergraduate Adult Development and Aging Psychology Course

**DOI:** 10.1093/geroni/igaa057.1999

**Published:** 2020-12-16

**Authors:** Kelly O Malley, Kirsten Graham

**Affiliations:** 1 New England GRECC, VA Boston, Boston, Massachusetts, United States; 2 Southern Utah University, Cedar City, Utah, United States

## Abstract

Active engagement in health policy by psychologists is vital to the well-being of the aging population; however, few feel prepared to engage in policy making or know how to get involved. A novel policy curriculum was developed and integrated into an undergraduate psychology course. N = 34 students completed assessments of policy knowledge and assignments designed to increase their skills, knowledge, and critical thinking about health policy. Students reported strong beliefs that psychological research can impact health policies and a desire to understand how to use research to inform policy; however, they reported less understanding of how policy is made. Preliminary evidence suggests students are interested in applying psychological research to policy processes; however, they do not know how to get involved. Policy education was easily integrated into the course, and further study is needed to determine students’ future engagement in health policy and change health policy skills.

